# *Tripterygium wilfordii* Hook F Treatment for Stage IV Diabetic Nephropathy: Protocol for a Prospective, Randomized Controlled Trial

**DOI:** 10.1155/2020/9181037

**Published:** 2020-06-10

**Authors:** Xu Lengnan, Zhao Ban, Wang Haitao, Liu Lili, Chen Aiqun, Wang Huan, Zeng Ping, Mao Yonghui

**Affiliations:** ^1^Department of Nephrology, Beijing Hospital, National Center of Gerontology, Institute of Geriatric Medicine, Chinese Academy of Medical Sciences, China; ^2^The MOH Key Laboratory of Geriatrics, Beijing Hospital, National Center of Gerontology, Institute of Geriatric Medicine, Chinese Academy of Medical Sciences, China

## Abstract

**Background:**

Diabetic nephropathy (DN) is a major cause of chronic kidney disease (CKD). There are no effective treatments to prevent or reverse the progression of DN. A preliminary study showed that *Tripterygium* glycosides from *Tripterygium wilfordii* Hook F (TwHF) with valsartan decrease proteinuria in patients with DN.

**Objectives:**

The objective of the present study is to investigate the efficacy and safety of *Tripterygium* glycosides from TwHF, a traditional Chinese medicine, for the treatment of DN. *Methods and Analysis*. This is a prospective, single-center randomized controlled trial. Seventy participants diagnosed with DN were recruited and randomized 1 : 1 to two groups: (1) angiotensin receptor blocker (ARB) combined with TwHF and (2) ARB-only. The treatment period is 48 weeks. The primary endpoint is 24 h proteinuria decreased level (reduction of 30% vs. baseline) after 48 weeks of treatment. The secondary endpoints are (1) all-cause and cardiovascular-related mortality, (2) development of ESRD (serum creatinine > 530.4 *μ*mol/L or estimated glomerular filtration rate (eGFR) < 15 mL/min/1.73 m^2^), (3) the need for renal replacement therapy, and (4) increased serum creatinine (2-fold higher than the baseline value or ≥442 *μ*mol/L, with confirmation of the initial results after 4 weeks). A health economics analysis will be carried out. *Discussion*. A meta-analysis of RCTs carried out in patients with stage 4 (Mogensen classification) diabetic CKD showed that TwHF combined with an ARB was more effective than an ARB alone when considering 24 h proteinuria and serum albumin, but with an increase in adverse event (AE) frequency of 8%. This is a prospective clinical trial that may provide information on a safe and effective novel method for the treatment of DN, especially for patients with macroproteinuria. *Ethics and Dissemination*. The protocol is approved by the ethics committee of Beijing Hospital (2016BJYYEC-059-02). The results will be disseminated through peer-reviewed publications and international conferences. This trial is registered with ChiCTR-IOR-17010623.

## 1. Introduction

Diabetic nephropathy (DN) is a microvascular complication of diabetes mellitus (DM) that may progress to chronic kidney disease (CKD) [1]. About 20%-40% of patients with DM develop DN [1]. The prevalence of diabetic kidney disease in the United States was 3.3% in 2005-2008 [2]. DN is a major cause of end-stage renal disease (ESRD) and is also the leading cause of mortality in patients with CKD, at 48 per 1000 patient-years at risk [1, 3, 4]. The prevalence of DM is approximately 11% in China, and 36% of the Chinese population have impaired glucose metabolism [5]. Type 2 diabetes mellitus (T2DM) represents the majority of cases of diabetes. It is estimated that type 1 diabetes accounts for less than 5% of diabetes cases in China [5, 6]. In our hospital, DN is the second underlying reason for dialysis, accounting for 35.9% of all cases of peritoneal dialysis and 25% of all cases of maintenance hemodialysis (MHD) [7, 8]. According to an etiology analysis of the MHD patients at our center over the previous 20 years, patients with DN tend to require renal replacement therapy earlier than non-DN patients. Those observations are supported by the general trend observed in China [9]. Therefore, DN currently is a major health, social, and economic burden in China.

Among patients with nephrotic proteinuria (proteinuria > 3 g/d), 75% will develop ESRD within 15 years, 50% within 10 years, and 25% within 6 years. In addition, most ESRD patients will die within 6 years [10, 11]. The degree of proteinuria is an independent risk factor for DN progression [12, 13]. Reducing proteinuria may be the key to slowing the progression of kidney damage and its accompanying complications [14, 15]. Unfortunately, the effect of renin-angiotensin-aldosterone system inhibitors is limited, especially for patients with massive proteinuria [16–20]. Therefore, a new treatment to effectively reduce overt proteinuria and delay or prevent the development of ESRD would be of great clinical significance.

The pathogenesis of DN is complicated [21, 22]. Recently, researchers have explored novel strategies to prevent the progression of DN using anti-inflammatory approaches, cytokine inhibition, and podocyte injury prevention [21, 23, 24]. A previous study by our group showed that large numbers of inflammatory cells infiltrate the renal tissue in DN patients, suggesting that inflammation plays a very important role in the development of DN [25]. Notably, new antidiabetic drugs, such as sodium-glucose cotransporter-2 (SGLT-2) inhibitors, have anti-inflammatory effects [26].

*Tripterygium wilfordii* Hook F (TwHF) extract is a traditional Chinese medicine (TCM) that has been used for nearly 40 years in the treatment of glomerulonephritis and during organ transplantation. Studies showed that TwHF can inhibit inflammatory cell-mediated response in kidney tissues, stabilize the permeability of the glomerular basement membrane, and protect podocytes in the presence of diabetes. TwHF is being increasingly used in the treatment of DN, and TwHF combined with angiotensin-converting enzyme inhibitors (ACEIs) and angiotensin receptor blockers (ARBs) represents a novel, potentially effective, and potentially safe regimen for the treatment of DN patients with proteinuria.

Yongchun and Shijun [27] evaluated the efficacy of TwHF extract compared to valsartan (an ARB) in the treatment of T2DM-induced DN. A total of 65 DN patients with proteinuria ≥ 2.5 g/d and serum creatinine levels < 3 mg/dL were enrolled in a 6-month, prospective, randomized controlled study. The mean proteinuria levels in the TwHF group was dramatically decreased at 6 months compared with the valsartan group (4.99 ± 2.25 vs. 2.99 ± 1.81 g/d; *P* < 0.01), with decreases (relative to baseline) at 1, 3, and 6 months of -32.9%, -38.8%, and -34.3%, respectively. In contrast, the proteinuria in the valsartan group was not significantly attenuated, and the changes in proteinuria levels at 1, 3, and 6 months were +1.1%, +10.1%, and -11.7%, respectively. The mean decrease in eGFR in the valsartan group was greater than that in the TwHF group, although not significant (26.4% vs. 13.7%, respectively; *P* = 0.067).

Multiple studies have indicated that ACEIs and ARBs combined with *Tripterygium* glycosides could enhance the treatment effects in DN patients via multiple mechanisms, but those mechanisms are not clear. Animal and *in vitro* studies have demonstrated that triptolide, a major active component of TwHF, has potent immunosuppressive, anti-inflammatory, and antiproteinuric effects [28]. Triptolide reduces established massive proteinuria and podocyte injuries and protects podocytes in patients with DM.

Unfortunately, there is still no standard treatment for DN and the efficacy and side effects of TwHF vary among individuals. Moreover, there is still no evidence on the long-term outcomes of TwHF. The study proposed here will confirm the efficacy and safety of TwHF. It will lay theoretical and practical foundations for the prevention and cure of DN.

## 2. Trial Objectives

The aim of present study is to investigate the efficacy and safety of *Tripterygium* glycosides from TwHF, a TCM, for the treatment and prognosis of DN.

## 3. Methods

### 3.1. Ethics

This study has been approved by the ethics committee of Beijing Hospital, Beijing, China (2016BJYYEC-059-02). Participants will be asked to sign the informed consent form. At any time, participants will be able to withdraw from the study. Data will be entered electronically. Original study forms will be kept locked at the study site and maintained in storage for a period of 3 years after the completion of the study. All data sets will be password-protected and only available to project investigators. Data sets cleaned and blinded of any identifying participant information, as well as the full protocol, will be available after the completion of the trial on request to the contacting author.

### 3.2. Data and Safety Monitoring Board (DSMB)

The Data and Safety Monitoring Board (DSMB) members are independent of the investigators and the steering committee. The DSMB is responsible for ensuring that the study participants are not exposed to unnecessary risks and that the study is conducted according to high scientific and ethical standards. The DSMB is responsible for advising on early termination of the study in the event of unexpected safety concerns or if treatment differences become apparent after the prespecified interim analyses. The committee will review the adverse events (AEs) after all participants are enrolled. The committee consists of two independent researchers with experience in nephrology. They will be unaware of treatment assignment unless they raise concerns and unblinding is judged necessary.

### 3.3. Study Design and Setting

This protocol has been written in accordance with the Standard Protocol Items: Recommendations for Interventional Trials (SPIRIT) statement. The planned study is a single-center, prospective, randomized, open-label, and controlled trial. The trial is an add-on trial as all participants will receive an existing treatment (ARB), but some will also receive the additional experimental treatment (TwHF).

We plan to recruit 70 participants who satisfy the inclusion and exclusion criteria. They will be randomly assigned to the experimental (ARB+TwHF) or control (ARB-only) group. After enrolment, all participants will be evaluated at baseline and every 2-4 weeks subsequently (depending on the efficacy and AEs of TwHF) for 48 weeks. Assessment of the TwHF concentration in the blood of the TwHF group participants will assist in the understanding of the metabolic types (fast, slow, or normal metabolic types), as well as the association of AEs with the blood levels of TwHF.

TwHF (10 mg/tablet) will be provided by De'ende Company, Zhejiang, China. De'ende Company will not participate in data collection, analysis, manuscript editing, or publication decisions. All drug expenses will be at the charge of the subjects. The study is sponsored by, and will be conducted at, the Department of Nephrology of Beijing Hospital, in addition to employees at the hospital having the responsibility for data analysis. The study has been registered in the Chinese Clinical Trial Registry (registration no. ChiCTR-IOR-17010623).

### 3.4. Participants

The participant population will be 40- to 70-year-old participants meeting the DN diagnostic criteria [29], consistent with Mogensen phase IV, and with 24 h urinary protein ≥ 1 g. They will also satisfy all the inclusion and exclusion criteria (Tables 1 and 2).

The reason we chose a proteinuria > 1 g/d as cut-off for including criteria was that according to our clinical experience, proteinuria of <1 g had little effect on prognosis, and treatment with TwHF might lead to more side effects.

### 3.5. Recruitment

Participants will be consecutively recruited via (1) the outpatient department of nephrology, (2) referral from other hospitals associated with our hospital, and (3) recruitment advertisements posted in our hospital. All recruitment materials will direct interested participants (and their families) to all doctors in our department and a research nurse. Potential participants who satisfy the inclusion and not the exclusion criteria will be informed by a renal physician.

A face-to-face meeting with the investigators will then be arranged for these participants. The research coordinator will discuss (1) the purpose of the study, (2) how the randomization process works, (3) a general overview of the two treatments (ARB+TwHF or ARB-only treatment) and outcome measures, (4) the potential risks and benefits associated with participation in the study, and (5) the required time commitment. The researchers will ensure that the participants understand that they can withdraw from the study at any time without any effect on the care that they will receive. Participants will decide whether or not to enter the trial. All participants will be required to sign the informed consent form.

We will use a variety of methods to reduce the dropout rate. First, all physicians in the department of nephrology will be involved in the study. Participants will be able to receive treatment and attend follow-up appointments involving any doctor and will be referred in a timely manner to the senior physician in charge of the study when necessary. Second, a research nurse will have the ultimate responsibility for all the follow-up work, including undertaking regular assessments, sending a reminder for the next follow-up appointment, and arranging and preserving the handwritten and electronic records of all participants. Finally, owing to the specificity of Chinese medical insurance and the policies of our hospital, the cost of diagnosis and treatment can only be reimbursed for participants who attend our hospital. These measures can ensure regular follow-up in our department.

### 3.6. Randomization

**Figure 1:**
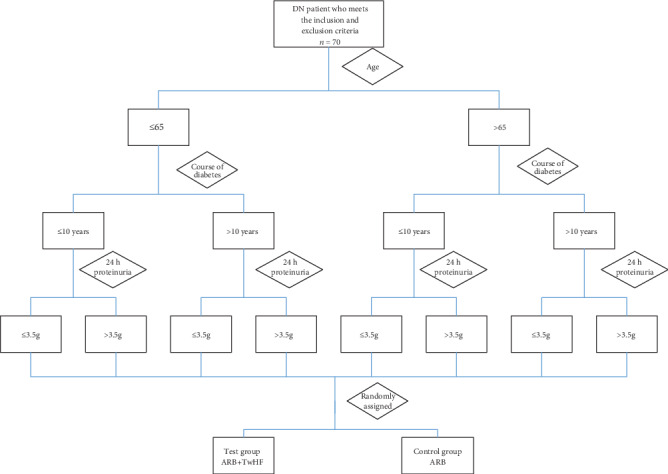
Stratified randomization method. DN: diabetic nephropathy; ARB: angiotensin receptor blocker; TwHF: *Tripterygium wilfordii* Hook F.

We will use stratified randomization. DN participants (*n* = 70) will be stratified based on the following factors: (1) age (≤65 or >65 years), (2) duration of diabetes (≤10 or >10 years), and (3) 24 h proteinuria (≤3.5 or >3.5 g). They will be randomly assigned to the experimental (ARB+TwHF) or control (ARB-only) group (Figure 1). The participants will be randomly assigned to the two groups according to computer-generated random numbers. The study will be open-label.

### 3.7. Treatment

**Figure 2:**
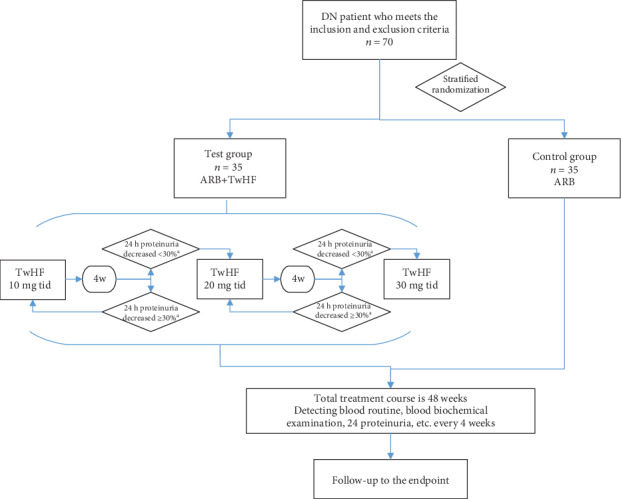
Study process. ^a^Compared with baseline. DN: diabetic nephropathy; ARB: angiotensin receptor blocker; TwHF: *Tripterygium wilfordii* Hook F.

In both groups, ARBs (unlimited category and dose adjusted according to blood pressure) will be used from baseline. A dose titration method will be used in the TwHF group. The starting TwHF dose will be 30 mg/d (thrice daily, 10 mg each time, 30 min after meals). After 2 weeks, 24 h proteinuria will be checked. If the proteinuria decreases by ≥30% compared to baseline or is <1 g, the TwHF dose will be maintained. If the proteinuria decreases by <30% compared to the baseline or is ≥1 g, the TwHF dose will be increased to 60 mg/d (thrice daily, 20 mg each time, 30 min after meals). Assessment of 24 h proteinuria will then be repeated every 2 weeks. If the proteinuria decreases by <30% compared to the baseline or is ≥1 g, the TwHF dose will be increased further to 90 mg/d (three times a day, 30 mg each time, 30 min after meals), which will be the maximum dose. After any dose adjustment, laboratory investigations, including 24 h proteinuria, will be conducted 1 week later. From the 12th week onwards, routine blood tests and 24 h proteinuria assessment will be conducted every 4 weeks. The total treatment duration will be 48 weeks. Treatment was discontinued if participants withdrew from the study for various reasons; for participants without events, treatment continued for 48 weeks. The whole study process is detailed in Figure 2.

During the study period, the following factors will be controlled: blood pressure (≤135/75 mmHg), blood glucose (fasting blood glucose < 8.0 mmol/L, 2 h postprandial blood glucose < 11.0 mmol/L, and glycated hemoglobin < 7%), and blood lipids (total cholesterol < 5.2 mmol/L and low‐density lipoprotein < 2.58 mmol/L). Treatments such as diuretics, albumin infusion, and symptomatic and supportive treatment can be used in the two groups, but 24 h proteinuria will have to be detected at least 3 days after completing any albumin infusion.

### 3.8. Endpoints

The primary endpoint is 24 h proteinuria decreased level (reduction of 30% vs. baseline) after 48 weeks of treatment. The secondary endpoints are as follows: (1) all-cause and cardiovascular-related mortality, (2) development of ESRD (serum creatinine > 530.4 *μ*mol/L or estimated glomerular filtration rate (eGFR) < 15 mL/min/1.73 m^2^), (3) the need for renal replacement therapy, and (4) increased serum creatinine (2-fold higher than the baseline value or ≥442 *μ*mol/L, with confirmation of the initial results after 4 weeks).

### 3.9. Other Endpoint

The focus of this study is clinical efficacy, but we also want to investigate whether we can decrease the cost of treatment borne by the participants. The main consideration will be the direct costs, comprising the annual costs to participants taking TwHF and dialysis and the cost savings of the ARB+TwHF participants compared to the ARB-only participants due to delayed dialysis. The above costs will be recorded by a research nurse at each follow-up session. At the end of the study, the study nurses will record all the medical expenses of the subjects and the annual expenses will be calculated. The discount rate used will be 3%.

### 3.10. Sample Size

Previous results regarding the treatment of DN with TwHF in China [27] indicated that 24 h proteinuria decreased (compared with baseline) 32.2-39.1% in the ARB+TwHF group and <10% in the ARB-only group. This indicates that the efficacy of ARB+TwHF is better than that of ARB-only treatment. Based on an *α* error of 0.05, a statistical power (1 − *β*) of 0.9, an estimated effect size (*δ*) of 1.2, and an estimated standard deviation of 2, the sample size estimate for this study is 58 participants (29 participants per group). Accounting for an attrition rate of 20%, the sample size is set to 70 participants.

### 3.11. Data Analysis

The demographic and clinical baseline data of the two groups will be compared using parametric or nonparametric test, as appropriate. A descriptive statistical analysis of the demographic and clinical baseline data will be presented, including 95% confidence intervals. The continuous data with normal distribution will be described using means ± standard deviation, and the count data with nonnormal distribution will be described using medians ± interquartile range.

We will use analysis of variance (ANOVA) or rank-sum test to compare the changes in the two groups between baseline and each follow-up point, using the baseline data as the reference. For the efficacy analysis, an intention-to-treat analysis approach will be used to reduce the influence of bias, increasing the validity of the results.

## 4. Trial Management and Monitoring

### 4.1. Monitoring and Processing of AEs

The termination criteria will be (1) noncooperation with the study personnel (noncooperation contrary to the research agreement, loss to follow-up, etc.) or termination for security reasons; (2) use of any immunosuppressants after starting the study; (3) serious adverse events (SAEs) (death, severe cardiovascular events, severe cerebrovascular events, or severe infections); (4) other adverse reactions or severe complications (severe gastrointestinal reactions, gastrointestinal bleeding, severe erythropenia, severe granulocytopenia, skin rash, etc.); (5) liver damage (increase in alanine aminotransferase or aspartate aminotransferase levels > 2‐fold compared to the upper limit of normal) or white blood cells < 3 × 10^9^/L (in these cases, initially, the TwHF dose will be reduced, and supportive treatment will be offered for 2 weeks; if the liver damage is reversed, TwHF treatment will recommence, but if not, the TwHF dose will be reduced further and all of the above parameters will be reviewed; if the liver damage is not reversed when the TwHF dose is reduced to 20 mg/d, the participant will cease treatment and be withdrawn from the study); and (6) participants from either group achieving the primary or secondary endpoints.

Previous research has indicated that TwHF tends to be well-tolerated and most reported AEs have been mild to moderate [27, 28]. In addition, previous research has suggested that the AEs of participants with renal insufficiency were reversible [27, 28]. In the planned study, TwHF dose titration will be used to reduce the occurrence of AEs. If serious adverse events occur and any of the termination criteria are met, withdraw and rescue medication will be offered.

If an AE occurs, a physician will provide timely treatment. In the meantime, the primary investigators (MYH and ZB) will be informed. The AE will be assessed and a treatment plan will be established. The research team will conduct follow-up on all participants and set follow-up interval according to the severity of their condition.

The following information will be recorded: the participant's signs, symptoms, and laboratory test results (in the original database and case report forms) and AEs occurring over time (including duration, severity, treatment, and outcomes). Investigators will ensure that the records are true, accurate, complete, timely, and legally compliant. In particular, the following system-specific AEs will be noted: (1) gastrointestinal system: loss of appetite, abdominal discomfort, nausea, vomiting, any other symptoms requiring supportive treatment, gastrointestinal bleeding, liver damage, and any other serious conditions; treatment will be stopped if required, and any internal bleeding will be stemmed, followed by hepatoprotective treatment, if required; (2) reproductive system: menstrual disorders, decreased sperm motility and number; (3) hematopoietic system: leukocyte and thrombocytopenia; and (4) others: skin rash, pigmentation, diabetes insipidus, or hearing loss. Symptomatic and supportive treatment will be provided if required, and treatment will be stopped if required.

## 5. Discussion

DN is a major cause of CKD and there are no effective treatments to prevent or reverse the progression of DN [1]. A preliminary study showed that *Tripterygium* glycosides from TwHF with valsartan decrease proteinuria in patients with DN [27]. Therefore, we present here a protocol for a RCT of TwHF in participants with DN. The objective of the present study is to investigate the efficacy of *Tripterygium* glycosides from TwHF for the treatment of DN.

DN has a complicated pathogenesis characterized by inflammation and invasion of the kidney by immune cells [21, 22]. The recent strategies for the management of DN include anti-inflammatory approaches, cytokine inhibition, and podocyte injury prevention [21, 23, 24]. TwHF is a TCM used for the treatment of kidney diseases and after organ transplant because of its immunomodulatory effects [30, 31]. Animal experiments and in vitro studies showed that the active components of TwHF can inhibit the expression of inflammatory cells and factors in kidney tissues, improve oxidative stress, stabilize the permeability of the glomerular basement membrane, and protect podocytes in the presence of diabetes [28].

A meta-analysis of RCTs carried out in patients with stage 4 diabetic CKD showed that TwHF combined with an ARB was more effective than an ARB alone when considering 24 h proteinuria and serum albumin, but with an increase in AE frequency of 8% [32]. A recent review indicated that TwHF-based therapies for CKD in mainland China were more effective in improving proteinuria, blood creatinine levels, and blood urea nitrogen levels than conventional Western medicine alone or other immunosuppressive regimens [33]. Meta-analyses highlighted that high-quality trials are still needed to confirm treatment efficacy [33, 34]. A preliminary Chinese study showed that TwHF combined with an ARB decreased proteinuria by about 30% throughout the treatment period, while the ARB alone had little-to-no effect on proteinuria [27]. In addition, the effect of TwHF in stage 1-3 diabetic CKD has been less studied than in patients with stage 4. The present study will only enroll participants with stage 1-3 diabetic CKD.

Some limitations may already be foreseen. This study focuses on the Chinese population, and caution should be used when applying the findings to patients of other ethnicities and because TCM is not recognized by many Western physicians. In addition, the health economic factors that will be considered are relatively simple. Only the direct costs will be considered, while the indirect costs will not be fully obtained. No cost-effectiveness analysis will be carried out.

Taken together, this is a prospective clinical trial that may provide information on a safe and effective novel method for the treatment of DN, especially for patients with macroproteinuria.

## 6. Study Status

The first participant was randomized on August 1, 2017. The recruitment will probably be completed by August 2020. Any changes to this original protocol will be reported in the final article.

## 7. Timeline and Dissemination

We anticipate that the study will be completed by January 31, 2020. We plan to communicate the results by presenting research abstracts at conferences and by publishing the results in a peer-reviewed journal. This study could bring a new therapy of DN. Participants will be provided a summary of the results as they become available. Finally, press releases of relevant findings will inform the general population.

## Figures and Tables

**Table 1:** Inclusion criteria.

1	Male or female of 40-70 years of age
2	Voluntary participation (signed an informed consent form before any study procedure)
3	Diagnosed in accordance with the diagnostic criteria for DN [29]; consistent with Mogensen stage IV: persistent large amounts of albuminuria (urinary albumin excretion rate > 200 mg/min) or proteinuria > 500 mg/d
4	24 h urinary protein ≥ 1 g
5	eGFR (calculated according to the CKD − EPI formula) ≥ 30 mL/min/1.73 m^2^ (equivalent to chronic kidney disease stage 1-3)
6	ARB use before and stable condition (no AEs and drug changing)

DN: diabetic nephropathy; eGFR: estimated glomerular filtration rate; CKD-EPI: Chronic Kidney Disease Epidemiology Collaboration; ARB: angiotensin receptor blocker; AEs: adverse events.

**Table 2:** Exclusion criteria.

1	Type 1 or secondary DM
2	Allergy to TwHF
3	Using any immunosuppressive agents
4	No reliable contraceptive is being used
5	Impaired liver function: ALT or AST levels ≥ 2‐fold the upper limit of normal
6	White blood cells < 3 × 10^9^/L
7	Severe and refractory hypertension (blood pressure > 180/100 mmHg)
8	Acute infection < 1 month prior to the study
9	Increase in serum creatinine during the study of ≥50% compared with baseline, inability to control severe hyperkalemia (serum potassium > 6.0 mmol/L) during past, or contraindications for ARB use (e.g., bilateral renal artery stenosis)
10	Serious cardiac or cerebrovascular event (heart failure, angina, myocardial infarction, cerebral infarction, cerebral hemorrhage, etc.) < 6 months prior to the study
11	Risk factor for poor compliance such as nonresident or without health insurance (at the investigators' discretion)
12	Participation in other clinical drug trials < 6 months prior to the study
13	Any medical practitioners deem that the trial is not suitable for the participant, for reasons such as cancer.

DM: diabetes mellitus; TwHF: *Tripterygium wilfordii* Hook F; ALT: alanine aminotransferase; AST: aspartate aminotransferase.

## Data Availability

The data used to support the findings of this study will deposite in the Chinese Clinical Trial Registry repository (Trial registration: ChiCTR-IOR-17010623).

## Abbreviations

ALT: Alanine aminotransferase
ACEI: Angiotensin-converting enzyme inhibitor
ARB: Angiotensin receptor blocker
AST: Aspartate aminotransferase
CKD-EPI: Chronic Kidney Disease Epidemiology Collaboration
CYP: Cytochrome P450
DSMB: Data and Safety Monitoring Board
DM: Diabetes mellitus
DN: Diabetic nephropathy
eGFR: Estimated glomerular filtration rate
ESRD: End-stage renal disease
ICMJE: International Committee of Medical Journal Editors
IDF: International Diabetes Federation
MHD: Maintenance hemodialysis
SGLT-2: Sodium-glucose cotransporter-2
SPIRIT: Standard Protocol Items: Recommendations for Interventional Trials
TwHF: *Tripterygium wilfordii* Hook F
T2DM: Type 2 diabetes mellitus.

## Additional Points

*Strengths and Limitations of This Study*. (i) This is a prospective clinical trial that may provide information on a safe and effective novel method for the treatment of DN, especially for patients with macroproteinuria. (ii) This study focuses on the Chinese population, and caution should be used when applying the findings to patients of other ethnicities. (iii) The health economic factors that will be considered are relatively simple: only the direct costs will be considered, while the indirect costs will not be fully obtained, which will affect the results. No cost-effectiveness analysis will be carried out; if a cost-utility analysis was used, better results could be obtained. *Provenance and Peer Review*. Not commissioned; externally peer reviewed. *Open Access*. This is an Open Access article distributed in accordance with the Creative Commons Attribution NonCommercial (CC BY-NC 4.0) license, which permits others to distribute, remix, adapt, build upon this work noncommercially, and license their derivative works on different terms, provided the original work is properly cited and the use is noncommercial. See: https://creativecommons.org/licenses/by-nc/4.0/.
